# Child and parent perspectives on healthier side dishes and beverages in restaurant kids’ meals: results from a national survey in the United States

**DOI:** 10.1186/s12889-017-4610-3

**Published:** 2017-07-25

**Authors:** Eleanor T. Shonkoff, Stephanie Anzman-Frasca, Vanessa M. Lynskey, Grace Chan, Meaghan E. Glenn, Christina D. Economos

**Affiliations:** 1 ChildObesity180, Tufts University, Boston, MA USA; 2 Jacobs School of Medicine and Biomedical Sciences, University at Buffalo, Buffalo, NY USA

**Keywords:** Nutrition, Restaurants, Children, Kids’ meals, Fruits/vegetables, Side dishes, Beverages, Restaurant menus, Parents

## Abstract

**Background:**

Children frequently consume foods from restaurants; considering the quick-service sector alone, 1/3 of children eat food from these restaurants on a given day, and among these consumers, 1/3 of their daily calories come from fast food. Restaurant foods and beverages are second only to grocery store foods and beverages in their contribution to total energy intake of U.S. 4- to 11-year-olds. Shifting their restaurant consumption in healthier directions could have a positive impact on child health. In 2014 this study examined self-reported child receptivity and parent awareness of child receptivity to ordering a fruit or vegetable side dish instead of French fries; and milk, water, or flavored water instead of soda/pop with a kids’ meal when eating out. Child receptivity to side dishes was compared between 2010 and 2014.

**Methods:**

An online survey was administered by Nielsen via their Harris Poll Online to a national panel of 711 parents and their 8- to 12-year-old child, as part of a larger study. Frequencies, logistic regressions, t-tests, chi-square tests, and percent agreement were used to evaluate child likelihood of ordering certain side dishes; receptivity to healthier side dish and beverage alternatives; changes in receptivity to healthier sides across years; and parent awareness.

**Results:**

A majority of children said they were likely to order a meal with a vegetable (60%), fruit (78%), or French fry (93%) side dish. They were receptive to receiving a fruit or vegetable (FV) side dish instead of French fries (68%); or milk, water, or flavored water instead of soda (81%) with their restaurant kids’ meal. Liking/taste was the most common reason for children’s feelings. Child receptivity to a FV side dish instead of French fries was high in both years and significantly higher in 2014 (*t* = −2.12, *p* = 0.034). The majority of parent and child reports of child receptivity were concordant (85%).

**Conclusions:**

These national survey results indicate that children are receptive to FV side dishes and healthier beverage options with their restaurant meals. Their receptivity has remained high in the recent past, and parents are aware of child receptivity. An opportunity exists for restaurants to leverage child receptivity to healthier sides and beverages by providing and promoting healthy options.

## Background

Children frequently consume foods from restaurants; considering the quick-service sector alone, 1/3 of children eat food from these restaurants on a given day [1, 2], and among these consumers, 1/3 of their daily calories come from fast food [3]. Restaurant foods and beverages are second only to grocery store foods and beverages in their contribution to total energy intake of U.S. 4- to 11-year-olds [1–3]. Restaurant food and beverages have been linked with overconsumption of calories, sodium, total fat, saturated fat, and sugar-sweetened beverages as well as under-consumption of milk, fruits, vegetables, and vitamins A and C in children [2, 4–7]. At full-service and quick-service restaurants, main dishes for children are often bundled with beverages and side dishes (referred to herein as “kids’ meals”), which may negatively affect nutritional quality of the meal when beverages and side dishes are high in calories and low in nutrients [6, 8–10]. Many kids’ meals at leading restaurant chains do not provide healthy side dishes like fruits and vegetables by default, making it difficult to meet recommended calorie, fat, saturated fat, and sodium limits for restaurant kids’ meals [11, 12].

Given the frequency with which children eat at restaurants, the setting offers an opportunity to expose children to healthy foods and to shift eating patterns to improve their healthfulness. Recent evidence suggests that some restaurants are offering a larger number of healthy options on children’s menus than in the past, with menu additions that are lower in calories, fat, and sodium and/or higher in fruits and/or vegetables [4, 13]. These healthier offerings could lead to healthier ordering patterns. In one full-service restaurant chain, the kids’ menu was redesigned to include more meals that met nutrition standards for the National Restaurant Association’s Kids LiveWell program; fries and soda were removed; and default side dishes became healthier options. After the new menus were introduced, child orders for fruits, vegetables, milk, and juice increased; and orders for French fries and soda decreased [14]. These healthier ordering patterns were present more than one and 2 years after the menu change, and restaurant revenue continued to grow over that time [14, 15]. While some restaurants are beginning to emphasize healthier options for children, such as removing sugar-sweetened beverages from kids’ menus, the majority of kids meals are still unhealthy [12, 16]. Increased availability and salience of healthy options may be avenues for improving child dietary intake in restaurant settings, but relatively little is known about parent or child receptivity to such changes.

Parents can play a key role in steering children toward healthier options in restaurants. Parenting style and parent’s own dietary intake have been associated with child fruit and vegetable consumption [17, 18]. Shared parent-child responsibility for ordering restaurant meals has been linked to lower-calorie orders for 3- to 12-year-old children, compared to child-directed ordering decisions [19]. Yet because parents consider child preferences when making food purchases, they may hesitate to purchase food they think their child will not like, with low-income parents being especially hesitant to spend money on food they expect to be wasted [20–22]. Additionally, parents may assume that their child is unwilling to accept healthier menu options when they may, in fact, be willing.

The success of future efforts to promote healthy eating in restaurants may hinge on children’s receptivity to healthier alternatives and parent willingness to purchase those healthier options. Thus, information on both child receptivity and parent awareness of that receptivity is needed. In a national survey of 8- to 12-year old children conducted in 2010, a majority of children reported that they would accept a fruit or vegetable side dish instead of French fries with their restaurant meal [11]. An updated and expanded assessment of children’s views is warranted given changes in the broader food environment over the last several years, including new school meal nutrition standards, the removal of soda/pop from some restaurant children’s menus, and other large-scale public health efforts related to childhood obesity [16, 23–25]. This study therefore had three aims: (a) to conduct an updated assessment of children’s receptivity to healthier sides in 2014 and add an assessment of their receptivity to healthier beverages; (b) to compare new findings to children’s perspectives on healthier side dishes in 2010; and (c) to assess concordance between parent and child reports of children’s receptivity to healthier side dishes and beverages.

## Methods

### Participants

Participants were 8- to 12-year-old children (*n* = 711) and one of their parents (*n* = 711). Parents were a subsample from a larger study of 1207 US parents of 5- to 12-year-olds. Parents who completed that online survey were asked if their child was between 8 and 12 years; if the child was in that age range, the child was invited to take a survey as well. Parents gave informed consent and permission for their child to participate, and children gave minor assent before beginning the survey. Parents received instructions that they could help their child complete the survey, if needed, but it was made explicit that answers should be the child’s alone; children were given the same instructions. Recruitment procedures and the current analytic sample are described in more detail below. All human subjects procedures were approved by the Tufts University Institutional Review Board.

### Procedure

Researchers with Tufts University developed a survey and commissioned Nielsen Holdings N.V. (New York, NY) to administer it online within the United States in July 2014, via their Harris Poll Online (HPOL). In addition to demographics, the survey contained 12 questions focused on habits, preferences, and knowledge related to eating in restaurants. The participant sample was obtained from the HPOL opt-in panel of millions of respondents. Invitations for the HPOL panel were emailed to a stratified random sample identified as US residents 18 years or older with a 5- to 12-year-old child in the household. Respondents were invited to participate in the survey through password-protected email invitations. The HPOL panel was recruited through hundreds of sources using diverse recruitment methods to minimize selection bias. Unless otherwise stated, the sample of interest is all children recruited and surveyed through this process in 2014 (*n* = 711).

Two items assessed how frequently children had restaurant food or take-out. Among those children reporting ever eating food from a restaurant, additional items assessed how often they ordered from the kids’ menu, their feelings about healthier side dishes and beverages and how likely they would be to order them (Table 1). Children who indicated that they would be either happy or unhappy to receive healthier sides and/or beverages were asked to explain their responses; those who answered “neither happy nor unhappy” were not. Parent awareness of child willingness to order healthier options was assessed with 2 items (Table 1). Parents also provided information about child dietary intake of fruits and vegetables using two items that have been previously validated against adolescent self-report [26]. Parent education was reported and collapsed into three categories: 1 = Completed high school or less; 2 = Associate degree, job-specific training, or some college; 3 = Four-year college degree (ex. BA) or more. Parents provided household income before taxes, which was used to calculate child eligibility for free or reduced price school meals following program eligibility criteria for 2013-2014 [27].

**Table 1:** Parent and child survey items for orders, receptivity to healthier alternatives and restaurant frequency

Question	Response options
Child	
How likely would you be to order or ask for a restaurant meal if it came with each of the following as a side dish? (a) Vegetables such as a salad, green beans, or carrots (b) French fries (c) Fruit, such as apple slices, orange slices, or a fruit cup	1 = Very unlikely to 4 = Very likely
How happy or unhappy would you be if your restaurant meal came with a vegetable or fruit side dish, but not French fries?^a^	1 = Very unhappy to 5 = Very happy
How happy or unhappy would you be if your restaurant meal came with milk, water, or flavored water, but not soda or pop?^a^	1 = Very unhappy to 5 = Very happy
How often do you eat food at a restaurant? This includes fast food restaurants (such as McDonald’s or Burger King), sit-down restaurants (such as Applebee’s or Chili’s), or a local restaurant in your neighborhood (such as a coffee shop or pizza place).	1 = Never to 6 = 4 or more times a week
How often do you eat take-away from a restaurant? This includes fast food restaurants (such as McDonald’s or Burger King), sit-down restaurants (such as Applebee’s or Chili’s), or a local restaurant in your neighborhood (such as a coffee shop or pizza place).	1 = Never to 6 = 4 or more times a week
Parent	
How happy or unhappy would your child be if [(his/her)] restaurant meal came with a vegetable or fruit side dish, but not French fries?	1 = Very unhappy to 5 = Very happy
How happy or unhappy would your child be if [(his/her)] restaurant meal came with milk, water, or flavored water, but not soda or pop?	1 = Very unhappy to 5 = Very happy

^a^Children were asked to explain their feelings in open-ended format unless they answered “neither happy nor unhappy”

In 2010, a separate sample of 8- to 18-year-old children (*N* = 1178) was recruited via HPOL to complete an online survey covering several topics in addition to restaurant preferences, as reported previously [11]. In addition to the aforementioned dataset, this 2010 sample was used for between-year comparisons reported herein. For that sample, survey invitations were emailed to a stratified random sample identified as U.S. residents ages 13 to 18 and to a stratified random sample identified as U.S. residents ages 18 years or older with an 8- to 17-year-old child in the household. Parents did not complete surveys in 2010. For comparisons between years (i.e. 2010 vs. 2014), analytic datasets were selected to ensure parallel inclusion criteria, given differences in the ages of the children studied and skip patterns used across the two samples. To arrive at two consistent samples of 8- to 12-year-old children who reported ever ordering kids’ meals, the 2010 data were restricted to only the 8- to 12-year-old participants, and the 2014 data were restricted to only those participants who reported ever ordering restaurant kids’ meals. This latter restriction was needed because questions about receptivity and willingness were only asked among children who reported ever ordering kids’ meals in 2010.The sample sizes for between-year comparisons were: *N* = 509, 2010 dataset; *N* = 622, 2014 dataset.

### Data analysis

Statistical analysis was conducted using SAS 9.2 (Cary, NC). Sampling weights were provided by Nielsen Holdings based on parent age, sex, race, ethnicity, education, region, and household income using a RIM (Random Iterative Method) weighting process, in which each respondent was given a single weight value. The individual weight values were capped based on standard parameters by sample size to limit any extreme weight or outliers. Sampling weights were incorporated into all analyses, and corresponding analytic methods were used (e.g., Proc Surveyfreq, Proc Surveylogistic). Sampling weights were based on all cases from the overarching study (US parents of 5- to 12-year-old children) rather than the subsample in the current study. Because this could affect accuracy of estimates of variance, a sensitivity analysis was conducted examining whether estimates of variance differed when analyzing the subsample using subpopulation commands on the larger dataset (i.e., “domain” function in SAS) versus using a subset of the data containing only the cases of interest [28, 29]. This process revealed that standard errors differed for correlations but not logistic regressions. Thus, correlations reported below were calculated using regressions on responses standardized to a mean of 0 and standard deviation of 1 using the “domain” statement. Logistic regressions reported below were conducted on the subsample only using survey analytic methods without the “domain” statement (e.g., Proc SurveyLogistic).

The first study aim was to examine children’s views about healthier options in restaurants in 2014. Children’s responses to how likely they were to order a meal that came with a fruit, vegetable or French fry side were each collapsed into two categories. Answers of 3 (“likely”) and 4 (“very likely”) were coded as “likely”, and answers of 2 (“unlikely”) and 1 (“very unlikely”) were coded as (“not likely”). Frequencies were calculated, and logistic regressions were used to test differences in likelihood by demographics and frequency of ordering from restaurants (i.e., dine-in or take-away). Children’s responses to how receptive they would be to receiving a fruit/vegetable side dish instead of French fries or to receiving milk/water/flavored water instead of soda/pop as their beverage were collapsed into two categories. Answers of 3 (“neither unhappy nor happy”), 4 (“somewhat happy”), and 5 (“very happy”) were coded as “receptive”, and answers of 2 (“somewhat unhappy”) and 1 (“very unhappy”) were coded as “not receptive”. Frequencies were calculated, and logistic regression models were used to test differences in receptivity by demographics and restaurant frequency (i.e., dine in or takeaway). Open-ended responses were coded into categories (see Table 2) by two researchers using an adapted version of the coding scheme used previously; a third coder resolved any discrepancies. Approximately 1.4% of responses were removed from the analysis because the participant’s answer was inconsistent (e.g., children provided reasons that they were unhappy after indicating they would be happy). Frequencies were calculated for each category.

**Table 2:** Demographic characteristics of families in the 2010 and 2014 samples used in the current study

	2010 (*n* = 509)		2014 (*n* = 711)	
Characteristic	Weighted freq.	Weighted %	Weighted freq.	Weighted %
Gender (female)	207.99	40.28%	350.64	49.21%
Child race/ethnicity				
Non-Hispanic white	334.84	64.84%	456.76	64.10%
Non-Hispanic black	74.72	14.47%	75.23	10.56%
Non-Hispanic Asian	6.03	1.17%	40.33	5.66%
Hispanic	78.81	15.26%	134.23	18.84%
Other	21.98	4.26%	6.00	0.84%
Free- or reduced-price meal eligibility for child				
No	--	--	490.35	71.30%
Yes	--	--	197.38	28.70%
Annual household income				
< $25,000	--	--	98.39	13.81%
$25,000-49,999	--	--	139.9	19.63%
$50,000-74,999	--	--	125.49	17.61%
$75,000-99,999	--	--	114.2	16.03%
≥ $100,000	--	--	209.74	29.44%
No answer	--	--	24.83	3.48%
Parent respondent education level				
High school or less	--	--	233.83	32.82%
Associate, training, some college	--	--	236.67	33.21%
College or more	--	--	242.05	33.97%

*Note:* Frequencies are weighted to be nationally representative

The second aim was to compare children’s receptivity to FV side dishes instead of French fries in 2010 versus 2014. Receptivity to healthier beverages was not assessed in the 2010 survey, and therefore no comparison to 2014 could be made. A *t*-test was used to compare means on the uncollapsed receptivity variable between years. Rao-Scott chi-square tests of independence were used to compare whether the proportion of children endorsing each reason for their feelings differed by year (i.e., 2010 and 2014).

The third aim was to assess correspondence between parent and child reports of child receptivity to a FV side instead of French fries; and milk, water, or flavored water instead of soda in 2014. Parent responses for how happy their child would be were collapsed using the same rules described above. Frequencies were calculated to examine percent agreement between parent and child reports.

## Results

### Participant characteristics and measurement properties

The weighted child sample from 2014 (*n* = 711) had an average age of 9.9 years (SE = 0.1) and was 49.2% female (SE = 2.7), 64.1% (SE = 2.8) non-Hispanic white, and 28.7% eligible for free- or reduced-price school meals (see Table 2). The sample of these children’s parents was 42.5 years old (SE = 0.5), 58.0% female (SE = 2.7), 34.0% had some college or more, and 33.4% had an annual household income of less than $50,000 (Table 2). The weighted sample of children from 2010 had similar age (M = 10.0. years, SE = 0.1), gender (40.3% female), and ethnic/racial distributions (Table 2). Supporting the validity of items used in the present study, two previously-validated items on parent-reported frequency of child fruit/vegetable consumption were positively correlated with parent assessment of child receptivity to healthier side dish alternatives (fruit consumption, *r* = .26; vegetable consumption, *r* = .31; *p*s < .0001). Child receptivity to a FV side was also positively correlated with parent report of child fruit and vegetable consumption (*r* = .29, *r* = .31, respectively, *p*s < .001).

### Child receptivity to healthier side dishes and beverages in 2014

**Fig. 1:**
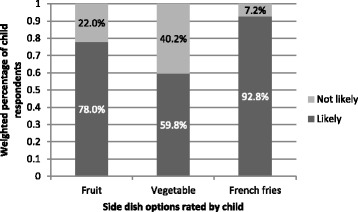
Weighted percentages of children in 2014 who reported being likely to order each type of side dish with their restaurant meal

**Fig. 2:**
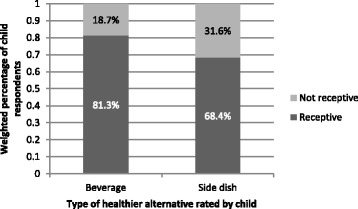
Weighted percentages of children in 2014 who reported being receptive to a healthier side dish or beverage instead of French fries or soda/pop

Most children (59.8%) reported that they would be likely to order a vegetable as a side dish; a larger percentage (78.0%) would be likely to order a fruit side, and nearly all (92.8%) would order a meal with a side of French fries (Fig. 1). Most children (68.4%) reported that they would be receptive to a FV side instead of fries, and 81.3% would be receptive to milk, water, or flavored water instead of soda/pop (Fig. 2). We tested whether child sex, child age, parent education, frequency of dining in restaurants, or frequency of getting take-out from restaurants was associated with (a) whether children were likely to order a fruit side dish, (b) whether children were likely to order a vegetable side dish, (c) whether children were likely to order a French fry side dish, (d) whether they were receptive to a FV side instead of French fries, and (e) whether they were receptive to a healthier beverage instead of soda/pop. Significant associations emerged for whether children were likely to order fruit, vegetable, and French fry side dishes but not for child receptivity to a healthier side dish or beverage alternatives (see Table 3). Girls were more likely than boys to report that they would order a vegetable side dish; younger children were more likely than older children to report that they would order fruit; children who ate in restaurants more frequently reported a higher likelihood of ordering both French fries and a vegetable side dish compared to those who ate in restaurants less frequently; and those who got take out more frequently reported being more likely to order a French fry side dish.

**Table 3:** Independent predictors of child-reported likelihood of ordering fruit, vegetable and French fry sides

	Likelihood of ordering a vegetable side dish			Likelihood of ordering a fruit side dish			Likelihood of ordering a French fry side dish		
	OR	95% CI	p	OR	95% CI	p	OR	95% CI	p
Gender (female)	**1.84**	**1.20, 2.84**	**0.01**	0.69	0.42, 1.13	0.14	0.97	0.44, 2.13	0.94
Age	0.96	0.83, 1.12	0.63	**0.75**	**0.63, 0.90**	**0.00**	1.14	0.87, 1.50	0.34
Dine in	**1.33**	**1.11, 1.61**	**0.00**	1.15	0.93, 1.42	0.20	**1.60**	**1.08, 2.37**	**0.02**
Take-out	1.17	0.99, 1.39	0.07	1.11	0.92, 1.34	0.28	**1.78**	**1.26, 2.51**	**0.00**

*Notes:* “Dine in” = Frequency of eating food in restaurants; “Take-out” = frequency of eating take away foods from restaurants; Bold indicates significance; A positive odds ratio corresponds to a higher likelihood of ordering the indicated item with increased standing on the independent variable (female; older; dine at restaurants more frequently; take out more frequently); These independent variables were not significant predictors of receptivity to healthier sides in lieu of French fries or healthier beverages in lieu of soda/pop

Children who felt happy or unhappy about healthier side dishes and beverages explained their feelings in open-ended format (side dishes, *N* = 505; beverages, *N* = 499). For children who answered this question, liking/taste was the most common explanation, accounting for 62.2% of those happy and 83.4% of those unhappy with a FV side dish instead of French fries, as well as 54.8% of those happy and 60.2% of those unhappy with the healthier beverage. Table 4 shows example child responses for each category. Other common reasons for children’s feelings were: health (25.1% of those happy about a FV side; 13.0% of those happy with a healthier beverage), habit (28.5% of those happy with a healthier beverage), and wanting a treat (13.2% and 32.5% of those unhappy about FV sides and beverages, respectively).

**Table 4:** Categories for coding open-ended responses about healthier sides and beverages with children’s restaurant meals

Categories	Sample responses
*For children who would be happy about the F/V alternative:*	
1. Liking/taste. Included the following:	
I like the taste of fruits and vegetables	“I LIKE FRUIT AND VEGGIES”
I like the taste of fruits	“I love fruit! More than French fries”
I like the taste of vegetables	“I like a lot of vegetables”
I don’t like/don’t prefer French fries	“do not like fries”
2. Health: Fruits and vegetables are healthy	“THE DOCTOR SAID IT WAS GOOD FOR ME AND MY DADDY SAID IT TOO”
3. Choice: I want choices/variety	“I would get to try something new”
4. Treat: Fruits and vegetables are a treat	“I like fruit and I can eat French fries at other times”
5. Habit: Child typically eats fruits/vegetables	“because i eat them all the time and like them”
6. Other	“because i don’t really care what side i get”
7. Don’t know	“sure”
*For children who would be unhappy about the F/V alternative:*	
1. Liking/taste. Included the following:	
I like French fries	“fries taste better”
I don’t like/don’t prefer fruit	“i don’t like fruit and fruit juice and i only like a few veggies”
I don’t like/don’t prefer vegetables	“I don’t like vegetables”
Other	“I only like some common fruits and only carrots for vegetables.”
2. Choice: I want to have choices	“I would like to have all of them.”
3. Treat: French fries are a treat	“I love French fries and I don’t get to eat them at home, so I would be upset.”
4. Habit: French fries are what I’m used to	“because when i go out to eat i like to get fries!”
5. Other	“i wouldn’t care”
6. Don’t know	“I want my fries”
*For children who would be happy about the beverage alternative:*	
1. Liking/taste. Included the following:	
I like milk	“i love milk”
I like flavored milk	“i like chocolate milk”
I like water	“I like to drink water”
I like flavored water	“I like flavored water”
I do not like/prefer soda or pop	“because i don’t like soda”
Other	“I would rather have juice”
2. Choice: I want to try something different	“try something new”
3. Habit: Child does not drink soda/soda not allowed	“I grew up not drinking soda.”
4. Other	“it is okay to have only water”
5. Don’t know	“Nice”
*For children who would be unhappy about the beverage alternative:*	
1. Liking/taste. Included the following:	
I like soda	“Soda is my favorite”
I do not like milk	“I hate milk.”
I do not like water	“i do not like milk or water” (also coded as “I do not like milk”)
I do not like flavored water	“... flavored water makes me feel sick”
Other	“I like to drink sweet tea with my food.”
2. Treat: Soda is a treat	“mom doesn’t buy coke at home so I like to get it when we go out.”
3. Habit: child typically drinks soda	“i always get soda at a restaurant”
4. Other	“I rather eat at home if I have to eat veggies and drink milk”
5. Don’t know	“because I want soda”

### Child receptivity to healthier side dishes in 2010 versus 2014

Children reported greater receptivity to a FV side dish instead of French fries in 2014 (M = 3.2, SD = 1.2) compared to 2010 (M = 3.0, SD = 1.2; *t* = −2.12, *p* = 0.034). The proportion of children endorsing each reason for their feelings did not differ significantly between years.

### Parent and child agreement on child receptivity to healthier side dishes and beverages

Most parents agreed with their children about whether the child would be receptive to a fruit/vegetable side dish instead of French fries (85.0%). Most (63.1%) agreed that the child was receptive, and 21.9% agreed that the child was not. Parents and children disagreed in the remainder of cases (15.0%) [parent thought the child was not receptive, but he/she was receptive (5.3%); parent thought that the child was receptive, but the child was not (9.7%)]. Similarly, most parents and children agreed about the child’s receptivity to milk, water, or flavored water instead of soda (86.5%): 73.5% agreed that the child was receptive, and 13.0% agreed that the child was not. The remainder disagreed (13.5%) [parent said the child was not receptive, but the child was (7.8%); parent said the child was, but the child was not (5.7%)].

## Discussion

To our knowledge, this study is the first to measure child receptivity to both healthier restaurant beverages and side dishes on a national scale. While a majority of children said they would order a meal with a vegetable, fruit, or French fry side dish, most were also receptive to a restaurant meal without French fries. A large majority of children were also receptive to a restaurant meal with water, flavored water, or milk instead of soda/pop. In addition, receptivity to healthier side dishes has remained high since 2010. Given the significant role that restaurants currently play in the diets of US children, shifting child beverage and side dish offerings to healthier options could have an important impact on overall calorie and nutrient intake. The restaurant setting offers an opportunity to expose children to healthy foods, potentially affecting dietary intake during the restaurant meal as well as possibly increasing children’s receptivity to fruits and vegetables in other settings. These findings demonstrate demand for healthier options in restaurants and support their inclusion and promotion on menus.

Children’s reported receptivity to healthier sides and beverages was high, in contrast to the resistance that may have been anticipated, and supports the inclusion of these items as defaults with children’s meals [14, 15]. Importantly, children in the current study were receptive to a meal *without* French fries or a soda but also reported a high likelihood of ordering French fries. One implication of these results is that changes to default menu offerings—i.e. making it easier and more automatic to receive healthier alternatives over these less-healthy side and beverage options—would be expected to improve children’s overall energy and nutrient intake to a greater extent than simply offering additional healthy options. Moreover, children who went to restaurants more frequently reported a higher likelihood of ordering both vegetable and French fry side dishes, similar to the previous study [19]. Consistent links between both frequency measures (both dine-in and take away) and willingness to order French fries could reflect exposure effects given that healthier children’s meals are still not the norm. Considering the literature on defaults and child restaurant orders, more frequent exposure could influence ordering habits [27, 28]. The implication is that healthier default options could eventually lead children to order healthy options out of habit; but unhealthy defaults could lead to habitual orders for and consumption of unhealthy foods and beverages as well. The acceptance of healthier options demonstrated in this study, combined with previous research supporting the power of default options in shaping behavior, suggests that large-scale implementation of healthy default options on children’s menus could create significant beneficial shifts in dietary intake of US children.

Taste was the primary reason children gave for their feelings about ordering healthier sides and beverages, followed by health and habit. These findings may reflect increased availability and accessibility of palatable, healthier options across settings (e.g. school, after school programs, restaurants) given that familiarity and repeated exposure help children learn to like and consume foods [4, 30, 31]. Recent changes to the food environment, such as more fruit and vegetable side dishes on US children’s menus and decreased soda consumption nationwide, may be helping to normalize healthier options, on restaurant menus, though these items have yet to become widespread [4, 32, 33]. Because taste was a strong deterrent for giving up French fries, restaurants might (a) use culinary creativity to improve the taste of healthier alternatives, (b) reduce portion sizes for food components that are overconsumed by US children, and (c) facilitate taste preferences for healthy foods, such as by offering taste tests [5, 34, 35]. Overall, the current findings suggest that while many children report liking the taste of healthier sides and beverages, French fries and soda may still hold more appeal for some children, underscoring the need to make healthy options easy (ex. defaults), plentiful (ex. frequent exposure) and to promote them in an engaging way.

The majority of parents in this study were aware that their child was receptive to healthier side dishes and/or beverages. This is in line with at least one previous study which found that parents were aware of children’s food preferences and took them into account when making purchases, but occasionally overrode child preferences for reasons like nutrition [22]. We would expect that parents who are aware of their child’s receptivity to meals without French fries or soda would be more willing to order healthier alternatives, though confirmation is an area for future research. Parents and families are important to include in efforts promoting healthy consumption for children given the links between family food practices, parent intake, and child intake [18, 36, 37]. Family-based health promotion efforts would likely result in greater improvements to child diets if healthy choices were the easier option within restaurants.

This study had strengths and limitations. To be eligible for the study, parents had to be members of Harris Poll Online, which could have biased the sample toward people with online access; however, the sample did include participants across a range of incomes. The sampling weights used in this analysis were for the overarching study rather than the current subsample. While they do not bring responses in line with demographics of 8- to 12-year-old US children specifically [38], analytic procedures were utilized that bring responses in line with US parent demographics. The large sample size weighted to match US demographics is a strength of the study. For aims comparing child responses across years, analytic datasets were restricted because of slightly different survey skip patterns and sampling methods in 2010 vs. 2014. This resulted in analysis of a slightly different sample for the between-year comparisons versus the other aims reported herein but allowed us to maintain consistent inclusion criteria across years in the former analyses. As with other self-reported data, social desirability bias is a possibility, but the inclusion of both parent and child responses is a strength; children may be less likely than adults to provide biased responses related to nutrition. Participants self-reported how they expected to respond in a restaurant situation, and it is unknown whether the high rates of reported receptivity would actually translate to healthier orders in restaurants. Some evidence suggests that purchase intentions better predict behavior when the choice is easy, such as when the product is well-known or the trade-offs of purchasing one product versus another are explicit; if these findings apply to children, the implication would be that self-reported intentions should predict ordering behavior, indicating that when children are familiar with all of the kids’ menu options, self-reported intentions should predict ordering behavior [39]. More research linking children’s self-reported receptivity to restaurant orders and consumption is warranted. The nascent literature evaluating children’s responses to healthier kids’ menu options is consistent with the current study’s conclusions and suggests willingness to accept healthier options in restaurants [14, 35, 40] with some variability [41]. Overall, findings support the idea that when healthy options are positioned as normative, such as being defaults or constituting a majority of the kids’ menu, child receptivity may be particularly likely to translate to healthier orders. Future work is needed to establish the link between child-reported receptivity and ordering behavior in restaurants. The questions and response options for children were similar to scales that have been validated for child populations [42, 43]; one of the questions was used successfully in a previous study [12]; and parent and child responses were correlated with previously-validated survey questions; and child self-reported receptivity to healthier options was correlated with parent report of fruit and vegetable intake, as described above, indicating convergent validity. However, additional measurement work on these items, including comparisons to objective measurements of ordering and intake in a restaurant, would provide further insights. Parents were given instructions that they could help the child complete the survey, if needed, which could have artificially inflated parent-child concordance. No outside verification of the independence of answers was possible, but instructions emphasized that children’s answers should be their own. Because child survey participation was an inclusion criterion, study results may not generalize to families in which the child would be unwilling to complete a survey with the parent.

## Conclusion

This study builds upon an emerging literature supporting the potential for healthier options to be accepted by children in restaurants [14, 15] and is the first to demonstrate that, on a national scale, children report receptivity to restaurant meals with milk, water, or flavored water instead of soda and continue to report receptivity to restaurant meals with a fruit or vegetable side dish instead of French fries. A majority of parents and children agreed that children would be willing to order these healthier options, and children identified taste as a primary factor influencing their feelings about restaurant food and beverage options. Considering the significant role of restaurant food and beverages in current diets of US children, further modifications to restaurant menus, such as incorporating palatable, healthier side dish and beverage options as defaults in kids’ meals, could have a significant impact on children’s calorie and nutrient intake. An opportunity exists for restaurants to leverage child receptivity to healthier sides and beverages by providing and promoting healthy options.
